# Usefulness of Probiotics in the Management of NAFLD: Evidence and Involved Mechanisms of Action from Preclinical and Human Models

**DOI:** 10.3390/ijms23063167

**Published:** 2022-03-15

**Authors:** Laura Arellano-García, María P. Portillo, J. Alfredo Martínez, Iñaki Milton-Laskibar

**Affiliations:** 1 Nutrition and Obesity Group, Department of Pharmacy and Food Sciences, Faculty of Pharmacy and Lucio Lascaray Research Center, University of the Basque Country (UPV/EHU), 01006 Vitoria-Gasteiz, Spain; laurai.arellano.garcia@gmail.com; 2 CIBER Fisiopatología de la Obesidad y Nutrición (CIBEROBN), Instituto de Salud Carlos III (ISCIII), 28222 Madrid, Spain; jalfredo.martinez@imdea.org (J.A.M.); inaki.milton@imdea.org (I.M.-L.); 3 BIOARABA Institute of Health, 01006 Vitoria-Gasteiz, Spain; 4 Precision Nutrition and Cardiometabolic Health, IMDEA-Food Institute (Madrid Institute for Advanced Studies), Campus of International Excellence (CEI) UAM+CSIC, Spanish National Research Council, 28049 Madrid, Spain

**Keywords:** probiotics, microbiota, liver steatosis, NAFLD, inflammation, parabiotics, postbiotics

## Abstract

The present review aims at analyzing the current evidence regarding probiotic administration for non-alcoholic fatty liver disease (NAFLD) management. Additionally, the involved mechanisms of action modulated by probiotic administration, as well as the eventual limitations of this therapeutic approach and potential alternatives, are discussed. Preclinical studies have demonstrated that the administration of single-strain probiotics and probiotic mixtures effectively prevents diet-induced NAFLD. In both cases, the magnitude of the described effects, as well as the involved mechanisms of action, are comparable, including reduced liver lipid accumulation (due to lipogenesis downregulation and fatty acid oxidation upregulation), recovery of gut microbiota composition and enhanced intestinal integrity. Similar results have also been reported in clinical trials, where the administration of probiotics proved to be effective in the treatment of NAFLD in patients featuring this liver condition. In this case, information regarding the mechanisms of action underlying probiotics-mediated hepatoprotective effects is scarcer (mainly due to the difficulty of liver sample collection). Since probiotics administration represents an increased risk of infection in vulnerable subjects, much attention has been paid to parabiotics and postbiotics, which seem to be effective in the management of several metabolic diseases, and thus represent a suitable alternative to probiotic usage.

## 1. Introduction

The prevalence of chronic metabolic diseases has been on the rise in the last decades, becoming a major health problem worldwide. Despite the amount of attention that has been paid to obesity, millions of deaths (up to 2 million by the year 2010) have also been attributed to liver diseases such as cirrhosis and hepatocellular carcinoma [1]. In this line, non-alcoholic fatty liver disease (NAFLD), also known as metabolic (dysfunction)-associated fatty liver disease (MAFLD), has become the most prevalent hepatic alteration in the last years [2]. Indeed, it is estimated that the prevalence of NAFLD is 20–30% in adults, and that this prevalence may well be higher in industrialized countries [3]. This hepatic condition includes relatively benign and reversible steatosis, characterized by excessive triglyceride (TG) accumulation in the liver, along with the more harmful stage known as non-alcoholic steatohepatitis (NASH), that can progress to cirrhosis or even hepatocellular carcinoma [4]. In this regard, besides the aforementioned excessive hepatic lipid accumulation leading to simple steatosis, further events such as inflammation, oxidative stress and fibrosis are also involved in the progression of the disease [5,6].

**Figure 1:**
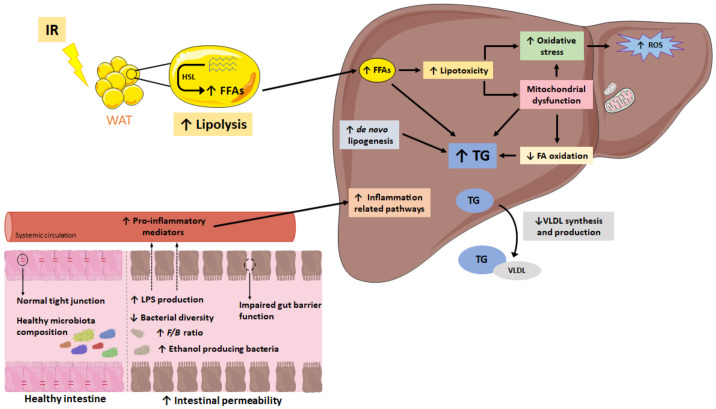
Simplified schematic representation of the events considered in the multiple-hit theory leading to NAFLD development. *F*/*B*: *Firmicutes*/*Bacteroidetes* ratio; FA: fatty acid; FFA: free fatty acid; HSL: hormone-sensitive lipase; IR: insulin resistance; ROS: reactive oxygen species; TG: triglyceride; VLDL: very-low-density lipoprotein; WAT: white adipose tissue. ↑: increase; ↓: decrease.

Due to the array of processes that have been identified to participate in NAFLD development, the once widely assumed “two-hit theory” has been replaced by the “multiple-hit theory” [6]. According to the latter, white adipose tissue insulin resistance plays a major role, impairing lipolysis and triggering inflammation. All these impairments result in a greater release of free fatty acids to the blood stream, which end up in the liver, thus contributing to excessive hepatic lipid accumulation. Additionally, this increased hepatic fatty acid deposition also results in lipotoxicity and subsequent mitochondrial dysfunction, which in turn increases reactive oxygen species (ROS) production and oxidative stress, and activates endoplasmic reticulum stress [6]. Moreover, gut microbiota alterations have also been described as contributing factors to NAFLD development. Impaired gut microbiota composition results in a greater production of pro-inflammatory cytokines, such as tumor necrosis factor α (TNF α) and interleukin 6 (IL-6), as well as microbial products with pro-inflammatory properties, including lipopolysaccharides (LPS) and unmethylated CpG DNA [7]. Moreover, increased intestinal permeability, resulting from altered tight junctions, leads to greater translocation of these pro-inflammatory mediators into circulation, which once reaching the liver, trigger the activation of pro-inflammatory pathways in the organ, thus contributing to the progression of NAFLD in NASH (Figure 1) [8]. As far as the causes leading to NAFLD development are concerned, excessive dietary fat and/or sugar intake (specially fructose) are considered among the main contributors [9,10]. Indeed, this kind of dietary pattern not only promotes excessive hepatic lipid accumulation (due to enhanced de novo lipogenesis and impaired mitochondrial fatty-acid oxidation), but it also induces liver inflammation, oxidative stress and mitochondrial dysfunction, all of which leads to the progression of hepatic damage [9,10]. Nevertheless, other factors such as food processing or polyphenol content can also have a role.

The high prevalence of NAFLD, as well as its potential implications in health, highlights the necessity for effective approaches in the prevention and treatment of this liver condition. However, since no specific treatment has been designed so far, conventional interventions based on dietary treatment and enhanced physical activity leading to body weight reduction are still widely prescribed [11,12]. One of the main reasons for using such an approach for NAFLD management relies on the higher prevalence of this hepatic condition in obese subjects. Indeed, according to recent data, it is estimated, that NAFLD is present in up to 50–90% of subjects featuring obesity [13]. Notwithstanding that the effectiveness of these approaches has been demonstrated, a common low adherence requires further therapeutic tools that may be prescribed as complementary or alternative treatments. In this scenario, the administration of probiotics for NAFLD has gained much attention, especially due to the involvement of gut microbiota alterations in the development of this liver alteration. By definition, probiotics are viable microorganisms that exert health benefits when consumed in sufficient amounts [14]. Thus, probiotic consumption may help normalize gut microbiota composition in patients with NAFLD, which in turn could result in improved gut barrier function and decreased pro-inflammatory cytokine production and release. Additionally, the recovery of gut microbiota eubiosis will also be helpful in restoring the production and levels of gut microbiota-derived metabolites with described health benefits, such as short-chain fatty acids (SCFA) [15].

In this context, the aim of this narrative review is to summarize the available evidence regarding probiotic usefulness in NAFLD prevention. In addition, the mechanisms of action described so far underlying the potential hepatoprotective effects of probiotics are also discussed. For this purpose, the first part of this manuscript is focused on the results obtained in preclinical studies (rodent models), whereas the second part summarizes the current evidence obtained from clinical trials. Additionally, limitations related to probiotic intake, as well as potential alternatives, are also discussed. With regard to the criteria followed to include or exclude articles in this narrative review, those using probiotics alone (single strain or mixtures) for NAFLD (not NASH) management, and studying variables such as liver fat content, liver histologic analysis (liver lipid content and/or inflammation) and transaminase levels, were selected. In the contrary, articles where none of these variables were analyzed or probiotics were administered along with other ingredients (unsaturated fatty acids or polysaccharides, for instance) were excluded. This article-selection task was carried out by two different persons.

## 2. Effects of Probiotic Administration (Single Strain and Mixtures) on NAFLD Prevention: Evidence from Preclinical Studies

When analyzing the potential usefulness of a molecule/compound in NAFLD prevention, both the molecule/compound and the stressor leading to the development of this hepatic condition are administered together. In preclinical studies, this liver alteration is commonly induced by using unbalanced diets characterized by a high content of fat and/or processed sugar. These feeding conditions not only result in an impaired nutrient intake, but they can also lead to an excessive caloric consumption. Moreover, diets lacking specific nutrients, such as choline-deficient diets, are also an effective approach when generating diet-induced NAFLD.

### 2.1. Preclinical Studies Using Single Strain Probiotics

Different studies have been carried out using a single-strain probiotic (Table 1). In general, the majority of these studies have addressed the effects of specific probiotic strains in animals challenged by diets leading to NAFLD. In this line, the administration of several probiotic strains (mainly *Lactobacillus* and *Bifidobacterium*) has been shown to be effective in reducing liver lipid accumulation under dietary conditions providing 40 to 65% of energy as fat [16,17,18,19,20,21,22,23,24,25,26,27,28,29,30,31]. Moreover, this effect is also maintained when excessive hepatic lipid accumulation is mediated by dietary conditions providing high sugar intakes (10 to 30% of energy as fructose). In this case, probiotic administration prevented liver lipid accumulation when standard diets were supplemented with fructose [32,33,34,35], as well as when high-fat and fructose intakes occurred concomitantly [33,34]. Furthermore, probiotic-administration-mediated liver fat accumulation prevention was also reported in a study in which NAFLD was induced using a choline-deficient diet [36]. It is worth noting that the aforementioned effects were described in both mice and rats receiving different probiotic doses (from 1 × 10^7^ to 1 × 10^10^ CFU/day) and during different treatment periods (from 4 to 42 weeks) (Table 1).

**Figure 2:**
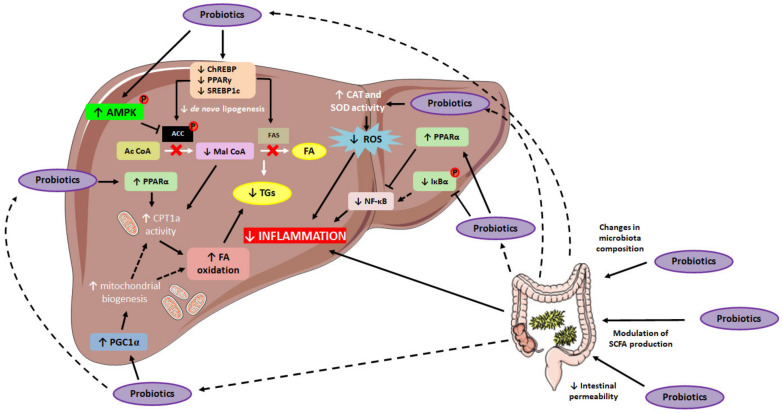
Schematic representation of single-strain probiotics-mediated effects in hepatic lipid accumulation. ACC: acetyl-CoA carboxylase; AMPK: AMP protein kinase; Ac Coa: acetyl CoA; CAT: catalase; ChREBP: carbohydrate-responsive element-binding protein; CPT1a: carnitine palmitoyltransferase 1a; FA: fatty acid; FAS: fatty acid synthase; Iκβα: NF-kappa-β inhibitor α; Mal CoA: malonyl CoA; NF-κB: nuclear factor kappa B; PGC1α: peroxisome proliferator-activated receptor gamma coactivator 1-α; PPAR-α: peroxisome proliferator-activated receptor α; PPAR-γ: peroxisome proliferator-activated receptor γ; ROS: reactive oxygen species; SCFA: short-chain fatty acids; SOD: superoxide dismutase; SREBP-1: Sterol regulatory element-binding protein 1; TG: triglyceride. ↑: up-regulation; ↓: down-regulation.

Regarding the mechanisms of action underlying the aforementioned probiotic-mediated effects on TG accumulation, several studies have described the down-regulation of lipogenesis [16,20,26,28,30,32,33,34,36]. In this regard, decreased liver gene and protein expressions, as well as diminished activation of de novo lipogenesis key-mediators such as fatty acid synthase (FAS) and acetyl-CoA carboxylase (ACC) have been reported [16,26,30,32,33,34]. Additionally, the down-regulation of transcriptional factors regulating the expression of these enzymes, including carbohydrate response element-binding protein (ChREBP), sterol regulatory element-binding transcription factor 1 (SREBP-1) and peroxisome proliferator-activated receptor γ (PPARγ) has also been described in studies using single-strain probiotics [16,20,30,32,33,34,36]. Thus, it seems that one of the mechanisms of action by which probiotic administration may prevent excessive hepatic lipid accumulation relies on their ability to reduce the expression and/or the activity of lipogenic mediators (Figure 2). Interestingly, the effects of single-strain probiotics in terms of hepatic lipid “output” have also been described. In this regard, several studies have highlighted the capacity of this intervention to modulate liver fatty acid oxidation, mainly by enhancing the activity of carnitine palmitoyltransferase-1a (CPT-1a), the enzyme that mediates the entrance of long-chain fatty acids into the mitochondria for their subsequent oxidation, and thus, is considered the rate-limiting step in long-chain fatty acid oxidation [16,20,33]. Moreover, some studies have also reported enhanced expression (gene and protein) of PPARα, which is known to control the activity of enzymes involved in mitochondrial β-oxidation (including CPT-1a) [20,30,33,36,38]. In addition, increased activation of AMP protein kinase (AMPK) has also been reported when administering single-strain probiotics [16,28,30,34,36]. In this line, the AMPK mediated ACC inhibition results in a lower production of malonyl-CoA, which is an inhibitor of CPT-1a, resulting in a greater activation of the latter. Finally, alongside the up-regulation of markers involved in lipid oxidation, higher peroxisome proliferator-activated receptor-γ coactivator 1α (PGC-1α) gene expression was reported under probiotic administration [26]. Since this transcriptional co-activator is considered as the master regulator of mitochondrial biogenesis [39], its up-regulation could result in an enhanced mitochondrial biogenesis, and thus, in an increased fatty acid oxidation.

Concerning the mechanisms of action underlying probiotic administration-mediated anti-inflammatory effects, several studies have described decreased gene and/or protein expressions of pro-inflammatory markers and mediators such as interleukin-1β, -4, -6 and -12 (IL-1β, IL-4, IL-6, IL-12), TNFα or nuclear factor kappa B (NF-κB) [16,17,18,26,28,30,32,33,36,37]. In addition, the aforementioned up-regulation of PPARα, which is known to negatively regulate NF-κB, may be involved in the anti-inflammatory effect of probiotic administration. Another process that is directly implicated in liver inflammation is the oxidative status of the organ, by cause of the tissue damage that occurs as a result of the imbalance between pro-oxidant production and antioxidant defense [40]. In this line, reduced liberation of ROS and production of lipid peroxidation and oxidative stress markers, such as malonaldehyde (MDA) or 4-hydroxynonenal (4-HNE), as well as enhanced activity of antioxidant enzymes, including catalase (CAT) and superoxide dismutase (SOD), have been described in several studies using single-strain probiotics [26,29,34,35,36]. Moreover, the up-regulation of the nuclear factor erythroid 2-elated factor 2 (Nrf2) pathway, including a greater expression of encoded antioxidant genes such as heme oxygenase 1 (HO-1), has also been described as a result of probiotic administration [36].

There are also a number of studies that have addressed the effects induced by probiotic administration on gut microbiota composition, microbial metabolite production and intestinal barrier integrity, all processes involved in NAFLD pathogenesis. Thus, the majority of the studies have reported that single-strain probiotic administration prevented diet-induced gut microbiota dysbiosis, mainly by increasing bacterial diversity and *Bacteroidetes* relative abundance and/or decreasing the *Firmicutes*/*Bacteroidetes* ratio [16,18,20,24,25,29,31,37]. In addition, the enhanced gene and protein expression of intestinal tight-junction markers, such as Occludin-1, Zonula Occludens-1 or Claudin, suggests that single-strain probiotic administration is an effective approach preventing high-fat and/or high-sugar diets induced gut barrier function impairment [17,20,32,36,37]. In this context, diet-induced impairment in gut microbiota composition, as well as the enhanced intestinal permeability, may well result in both a greater microbial production of LPS and its translocation to the liver. Indeed, once reaching the liver, LPS is known to bind receptors such as toll-like receptor 4 (TLR-4), releasing inflammatory mediators, and thus contributing to liver inflammation and NAFLD progression [13]. In this regard, the reduction induced by single-strain probiotic administration in TLR-4 expression may well account for the liver anti-inflammatory effect described for this intervention [17,30]. Finally, some authors have reported that the levels of SCFA in the cecum and in the liver are also modulated by probiotic administration. In this regard, it has been reported that the levels of acetate, a SCFA with known appetite modulating and hepato-protective properties [17,20,32,36,37,41,42,43], are increased after single-strain probiotic administration [23].

### 2.2. Preclinical Studies Using Probiotic Mixtures

As it has been demonstrated in studies using single-strain probiotics, the administration of probiotic mixtures as a preventive approach in rodent models featuring NAFLD has also been reported as effective. Thus, reductions in liver weight, fat accumulation and TG content, as well as decreased liver inflammation, have been described in studies using mixtures of different probiotic strains, administered in a wide range of doses and treatment periods (Table 2) [18,35,44,45,46,47,48,49,50,51].

The studies that analyze the effects of probiotic mixtures in NAFLD prevention are more limited than those conducted using single-strain probiotics. Interestingly, the described effects, as well as the involved mechanisms of action, seem to be similar. In this regard, the down-regulation of de novo lipogenesis, along with the upregulation of energy yielding pathways and fatty acid oxidation, seem to be among the mechanisms of action modulated by probiotic mixture administration that can prevent lipid accumulation in the liver [44,45,48,51]. Concerning liver inflammation, the administration of mixtures of probiotic strains effectively decreased the expression (gene or protein) and levels of pro-inflammatory markers and mediators, such as TNFα, myeloperoxidase (MPO), Il-6, Il-12, Il-1 β, interferon γ (Inf-γ), monocyte chemoattractant protein-1 (Mcp-1), inducible nitric oxide synthase (iNOS) and cyclooxygenase (COX) [18,44,45,48,49]. In addition, as occurred in studies using single-strain probiotics, it has been reported that probiotic mixtures decreased the expression of LPS and TLR-4 [45,48,49]. In this regard, LPS is known to interact with TLR-4, triggering cytokine cascades and inflammation [52] and, thus, their down-regulation might be regarded as having an anti-inflammatory effect.

In this context, and similarly to what has been described in studies using single-strain probiotics, the administration of probiotic mixtures seems to exert anti-inflammatory effects by modulating the NF-κβ pathway (Figure 2). Thus, the administration of probiotic mixtures not only down-regulates the protein expression of NF-κβ, but it also prevents its nuclear translocation, as suggested by the reduced phosphorylation found in its p65 subunit [45]. Furthermore, the administration of probiotic mixtures can also modulate this pathway by preventing the phosphorylation and subsequent degradation of the NF-κβ inhibitor (Iκβα), thus averting the nuclear translocation of NF-κβ [45]. Moreover, according to the reported results, the anti-inflammatory effects exerted by the administration of probiotic mixtures in the liver can be potentiated, at least partially, by the reduction in hepatic oxidative stress induced by this approach (Figure 2). In this regard, the up-regulation of enzymes with antioxidant capacity, such as CAT, SOD and glutathione peroxidase (GSH-Px), has been described, as well as a reduction in ROS levels and markers of lipid peroxidation including MDA [35,47].

Besides the aforementioned effects and underlying mechanisms of action, probiotic mixtures have also shown to effectively regulate an array of processes in the gut. For instance, several studies have reported a modulation of gut microbiota composition, resulting in an enhanced microbial diversity (enriched in beneficial commensal bacteria), as well as reduced *Firmicutes*/*Bacteroidetes* ratio values or enhanced relative abundances of microbial species such as *Bacteroidetes* or *Akkermansia* [45,46,47,48]. In addition, a modulation of the levels of microbial metabolites such as SCFA have also been described, which may be related to the aforementioned effects induced by probiotic mixture administration in gut microbiota composition. Thus, the enhanced abundances of butyrate and propionate, which are SCFA with well-known anti-inflammatory properties [41,43], have been described [46,47]. Finally, the administration of probiotic strain mixtures also seems to be an effective approach in terms of gut barrier function maintenance. In this line, this therapeutic approach resulted in increased gene and protein expression of intestinal tight-junction markers, including Zonula Occludens-1, Occludin-1 and Claudin-1 [44,45,46,48], which suggests an improvement in intestinal mucosa integrity, which in turn may prevent/reduce the delivery of pro-inflammatory mediators into the circulation.

Altogether, and based on the studies analyzed within this section, it could be concluded that the administration of probiotics, as single strains or mixtures, results in an effective intervention in diet-induced NAFLD prevention/management. Likewise, according to the published results, it may also be concluded that the effects produced by both types of interventions are similar, and that they are mediated by the same metabolic pathways. Therefore, it seems that the administration of a mixture of different probiotic strains does not represent an advantage compared to single-strain probiotic administration. Finally, it is worth mentioning that these effects have been described when the probiotics had been administered along with the stressors that lead to NAFLD development. Therefore, much attention should be paid to the analysis and interpretation of such data, since these experimental conditions are not likely to be reproduced in humans for obvious ethical reasons.

## 3. Effects of Probiotic Administration (Single Strain and Mixtures) in NAFLD Prevention: Evidence from Clinical Trials

The potential of probiotics for NAFLD management has also been investigated in humans. In this regard, the available studies addressing the effects of probiotics in NAFLD are more limited than those carried out in animals. Unlike preclinical studies, in which the preventive effect of probiotics on steatosis has been analyzed, studies in humans have addressed their therapeutic effects. In some studies, probiotics were combined with other compounds/molecules, thus creating a synbiotic, and consequently, the reported effects in NAFLD treatment cannot be attributed solely to probiotics. This represented a limitation when selecting suitable studies to be included in this narrative review article. Moreover, besides the probiotic treatment, the participants also received some sort of dietary advice and/or were encouraged to practice physical activity.

According to the majority of the clinical trials included in this review article, probiotic administration seems to be effective in the treatment of NAFLD (Table 3). Interestingly, and contrary to that observed in preclinical studies, most of this research has been conducted using probiotic strain mixtures, instead of single-strain probiotics. Decreased hepatic lipid content, reduced steatosis grade and lowered serum transaminase levels have been reported in studies using different probiotic strain combinations (including from 2 to 8 different probiotic bacteria strains), doses (from 5 × 10^8^ CFU/day to 22.5 × 10^10^ CFU/day) and administration periods (from 8 weeks to 12 months) in patients featuring NAFLD [53,54,55,56,57,58]. It is worth noting that these effects were described even in studies where all the participants (including the control group) received dietary advice (aimed at inducing body weight reduction), and also included physical activity programs or pharmaceutical treatment for further health alterations (statins and fibrates) [56,57]. In addition, similar hepatoprotective effects were also described in a study in which probiotics were administered mixed in a yogurt, instead as a supplement (capsule or sachet) [59]. Nevertheless, there are also studies in which the administration of probiotics did not result in the improvement of markers of liver injury in patients with NAFLD, despite the fact that the doses used and the administration periods were similar to those studies in which significant improvements were reported (Table 3) [60,61]. In this regard, according to Mohamed Nor et al. [60], the reduced sample size, along with the apparent higher variability of Malaysians’ gut microbiota composition (due to a more diverse dietary intake), may have influenced the obtained results. Moreover, the authors also pointed to a change in dietary fat observed in the group receiving the probiotic, which could have somehow blunted the potential beneficial effects of the probiotic intervention [60]. As far as the study carried out by Chong et al. [61] is concerned, differences in baseline characteristics between the probiotic and the placebo groups, as well as the impossibility to determine the participants’ NAFLD severity, were pointed out by the authors as potential factors influencing the outcomes of the study. Moreover, the participants in the studies in which no probiotic-administration-derived benefits in NAFLD were reported were older than in the rest of the studies. Since age-related variations in gut microbiota composition have been identified [62], it cannot be ruled out that this variable may have also influenced the outcomes of these studies.

Besides the aforementioned effects on liver TG accumulation, anti-inflammatory properties have also been described in studies addressing the effects of probiotic administration in NAFLD patients. In this regard, decreased circulating levels of pro-inflammatory mediators such as TNF-α, IL-1β or IL-6, as well as lowered hepatocyte ballooning and liver lobular fibrosis (assessed by histological analysis) have been observed [55,57]. Similarly, and in line with the outcomes found in preclinical studies, probiotic administration also resulted in gut microbiota modulation in patients with NAFLD. In this case, increased relative abundances of *L. acidophilus*, *L. rhamnosus*, *P. pentosaceus*, *B. lactis* and *B. breve* were found in obese NAFLD patients receiving a probiotic mixture (containing six different strains) for 12 weeks [56]. By contrast, none of the studies included in this review section have addressed the effects of probiotic administration on SCFA levels and/or intestinal integrity.

Based on the results reported in clinical trials, it could be concluded that in general terms, probiotic administration effectively improves markers of liver injury in patients with NAFLD. Indeed, the major effects that have been described to date, such as lower intrahepatic lipid content, decreased liver injury, as well as decreased circulating transaminase and pro-inflammatory cytokine levels, are compatible to those reported in preclinical studies. However, one of the main limitations of studies conducted in humans relies on the difficulty to obtain samples that may make it possible to investigate the mechanisms of action involved in these hepatoprotective effects. Notwithstanding that different non-invasive imaging techniques including ultrasound, computer tomography or magnetic resonance imaging have been demonstrated to be effective detecting liver fat infiltration, these are not appropriate to assess liver inflammation or fibrosis [63]. Furthermore, these techniques are not suitable to explore the pathways and mechanisms of action modulated by probiotic administration. In this regard, liver biopsies represent the gold standard to study hepatic inflammation and fibrosis, as well as the mechanisms of action involved in the effects mediated by probiotics. Unfortunately, since the procedures needed to obtain such samples happen to be very invasive, markers that can be more easily studied (such as serum transaminase or cytokine levels) are usually selected to elucidate the effectiveness of these approaches in NAFLD.

## 4. Limitations of Probiotic Administration and Potential Alternatives

According to the studies included in this review, as well the ones found in the literature that address the effects of probiotics on diseases other than NAFLD, probiotic administration represents an effective therapeutic tool for the management of an array of metabolic alterations including obesity, diabetes or dyslipidemia [64,65,66,67,68]. In this regard, besides the more “conventional” probiotics such as *Bifidobacterium* and *Lactobacillus*, much attention has also been paid to other microorganisms referred to as next-generation probiotics (NGP) as potential therapeutic approaches for NAFLD management. These “new” probiotics, resulting from improved culture methods, bioinformatics and next-generation sequencing, include such species as *Akkermansia muciniphila*, *Faecalibacterium prausnitzii*, *Eubacterium hallii*, *Propionibacterium*, *Bacteroides fragilis* and genus belonging to the Clostridia clusters IV, XIVa and XVIII [69]. For instance, lower *Akkermansia muciniphila* abundances have been related to metabolic disorders such as obesity and NAFLD [70,71]. Interestingly, the administration of this bacteria was found to be effective in ameliorating obesity and related metabolic disorders, but without affecting gut microbiota composition [65]. Moreover, the administration of heat-treated *Akkermansia muciniphila* was also shown to exert metabolic benefits, similar to those produced by the administration of viable bacteria [72]. In addition, it was reported that in NAFLD patients, the abundance of *Faecalibacterium prausnitzii* tends to be low. Since this bacteria is known to produce butyrate, its usefulness for NAFLD prevention was proposed [69]. Similarly, *Roseburia* spp. are butyrate-producing bacteria, and as in the case of *Faecalibacterium prausnitzii*, may be effective for NAFLD management. Indeed, it was reported that *Roseburia* spp. administration reduces hepatic steatosis and inflammation, mainly by restoring the gut microbiota environment and intestinal integrity [73]. Therefore, current available data suggest that the NGP may represent an additional therapeutic tool for NAFLD prevention and treatment.

Despite the aforementioned probiotic health benefits, their administration also involves some hazards, since this therapeutic approach is based on the administration of life/viable microorganisms to vulnerable subjects, which in turn results in an increased risk of systemic infection and/or immune system overstimulation [74]. In this line, much attention has been paid to the usage of parabiotics and postbiotics as alternative approaches to probiotics [75].

In the case of parabiotics, also referred to as paraprobiotics or ghost probiotics, these are usually obtained by inactivation of probiotic bacteria, mainly by means of thermic treatment [76]. In this regard, the efficacy of parabiotics relies on the molecules and compounds contained in inactivated bacterial cells, and not in their viability [77]. In comparison to probiotics (live bacteria), parabiotics represent several potential advantages, which include a lower risk of infection and antibiotic resistance acquisition/transfer, as well as an easier storage and handling [78]. Even though the available data regarding the usage of parabiotics in the management of different diseases are still scarce, it was described that administration of heat-inactivated probiotic bacteria (*Streptococcus thermophilus* MN-ZLW-002) is effective in preventing high-fat diet feeding induced body weight gain, insulin resistance and dyslipidemia in mice [79]. In addition, according to data reported in clinical trials, the continuous administration of fragmented *Lactobacillus amylovorus* CP1563 (heat inactivated, lyophilized and then milled) for 12 weeks significantly reduces whole body and visceral fat, ameliorates markers related to glycaemic control (reduced fasting blood glucose and insulin levels) and improves dyslipidemia (reducing blood TG, and total and LDL cholesterol levels) in subjects featuring class I obesity [80]. Indeed, in a recent systematic review addressing the efficacy of parabiotics in the prevention and treatment of different diseases, compared to probiotics, no significant differences were reported regarding the effectiveness of parabiotics in the majority of the preventive and treatment trials analyzed (86% and 69%, respectively) [81]. Thus, although the available evidence concerning parabiotic use as therapeutic approach is limited, the results reported so far suggests that overall, their efficacy is similar to that attributed to probiotics.

In respect to postbiotics, these encompasses a wide spectrum of non-viable bacterial products and cell components with potential bioactive activity in the host, including certain vitamins (A and K, for instance), bile acids, SCFAs, polyamines, branched-chain amino acids or components of bacterial cell wall, such as teichoic acids [77,82]. As occurs for parabiotics, data regarding the efficacy of postbiotics is still scant and mainly limited to preclinical studies. According to the studies that have been published so far, in older mice, the administration of lipoteichoic acid from heat-inactivated *Lactobacillus paracasei* D3-5 prevented high-fat diet feeding-induced metabolic dysfunction [83]. Similarly, the administration of the polyamine spermidine has been reported to effectively prevent high-fat diet feeding-induced body weight gain, liver lipid accumulation or insulin resistance in mice [84,85]. In these cases, the administration of the postbiotics resulted in the amelioration of gut microbiota dysbiosis and inflammation, as well as in the recovery of intestinal integrity [83,84,85].

Altogether, and despite the fact that further research is warranted, these data suggest that the administration of parabiotics and postbiotics may also prove effective in the management of certain diseases, as well as highlighting that the functionality of such compounds is beyond microbial viability.

## 5. Conclusions

The aim of the present narrative review article was to summarize the evidence available regarding the effectiveness of probiotic administration in NFALD management. In this context, studies conducted in rodent models have revealed that both the administration of single-strain probiotics, as well as the administration of probiotic mixtures, represent an effective approach in the prevention of this liver condition. Based on the magnitude of the observed effects, along with the described mechanisms of action, it could be suggested that compared to the usage of single-strain probiotics, the combination of different probiotic strains does not represent an advantage. In the case of studies conducted in humans, in the majority of them, probiotic administration also resulted in the amelioration of markers of liver injury in NAFLD patients.

With regard to the mechanisms of action underlying the effects that have been described so far, preclinical studies have demonstrated that probiotics (single or mixed strains) act in the liver, down-regulating lipid synthesis, activating lipid oxidation and down-regulating pro-inflammatory pathways, as well as in the gut, modulating microbiota composition, intestinal integrity and the production of microbial metabolites. As for clinical studies, data describing such mechanisms are scarce, mainly due to limitations in terms of obtaining samples. In this regard, metagenomics and metabolomics may represent a useful tool to better assess the effects of probiotic administration using samples such as blood, urine or feces. Similarly, further research is warranted in order to elucidate whether the administration of parabiotics or postbiotics constitutes a real alternative to the usage of probiotics for NAFLD management, and thus, to overcome the limitation that represents the administration of viable microorganisms to vulnerable subjects.

## Figures and Tables

**Table 1:** Preclinical studies (rodent models) addressing the effects of different single-strain probiotics on diet-induced NAFLD.

Reference	Animal Model	Experimental Conditions	Probiotic Treatment	Effects on Liver	Mechanisms of Action
[32]	Female C57BL/J6 mice 6-week-old	STD diet with 30% fructose in drinking water.	*L. rhamnosus* GG—LGG Daily administration Dose: 5.2 × 10^7^ CFU/bw g/d. Diluted in drinking water Treatment length: 8 w.	↓ Liver fat accumulation ↓ Liver TG content ↓ Serum ALT levels ↓ Liver inflammation	Down-regulation of lipogenic markers in the liver: ↓ Gene expression of *Acc*, *Fas* and *Chrebp*. Down-regulation of pro-inflammatory markers and mediators in the liver: ↓ Gene expression of *Il-1β*, *Il-8R*, *Tnfα* and *Il-12*. Decreased portal levels of LPS. Up-regulation of markers of intestinal mucosa integrity: ↑ Protein expression of Occludin-1 and Claudin-1.
[16]	Male C57BL/6 mice 4-week-old	HFD (60% energy from fat).	*L. rhamnosus GG*—*LGG* Oral daily administration Dose: 1 × 10^8^ CFU/day Treatment length: 13 w.	↓ Liver weight ↓ Liver fat accumulation ↓ Liver inflammation	Down-regulation of lipogenic markers in the liver: ↓ Gene expression of *Srebp-1* and *Ppar-γ*. Down-regulation of genes related to long-chain fatty acid uptake and lipoprotein synthesis: ↓ Gene expression of *Cd36* and *ApoB100*. Down-regulation of pro-inflammatory markers and mediators in the liver: ↓ Gene expression of *Il-6*, *Il-12*, *F4*/*80* and *Cd11b*. Modulation of gut microbiota composition: ↑ Proportion of *Bacteroidetes*.
[17]	Male C57BL/6 mice 4-week-old	HFD.	*L. paracasei* N1115 Oral daily administration Dose: 2.2 × 10^9^ CFU/mL diluted in normal saline (0.5 mL/day). Treatment length: 16 w.	↓ Liver fat accumulation ↓ Liver inflammation ↓ Liver fibrosis	Decreased content of hepatic inflammatory mediators (Tnfα and IL-1β). Down-regulation of pro-inflammatory markers and mediators in the liver: ↓ Gene expression of *Nf-κB*, *Tlr-4* and *Lps*. Decreased serum levels of liver fibrosis markers (MAO). Up-regulation of markers of intestinal mucosa integrity: ↑ Protein expression of Occludin-1 and Claudin-1.
[33]	Female C57BL/6N mice 6–8-week-old	STD diet with 30% fructose in drinking water.	*L. rhamnosus* Oral daily gavage Dose:1 × 10^9^ CFU/day. Treatment length: 5 or 12 w.	↓ Liver fat accumulation ↓ Liver TG, TC and VLDL content ↓ Liver inflammation ↓ Liver apoptotic cells	Down-regulation of liver injury protection markers: ↑ Gene expression of *Fgf21*. Down-regulation of pro-inflammatory markers and mediators in the liver: ↓ Gene expression of *Tnfα* and *Cxcl10*. Down-regulation of lipogenic markers in the liver: ↓ Gene expression of *Fas*, *Srebp1c* and *Scd1*. ↓ Protein expression of SREBP1c and ChREBP. Up-regulation of fatty acid oxidation markers in the liver: ↑ Gene expression of CPT1 and PPARα. Down-regulation of markers involved in hepatic ceramide content: ↓ Methylation of PP2AC.
[34]	Male C57BL/6N mice 8-week-old	HFD/F (65% energy from fat and 30% fructose solution).	*L. plantarum* NA136 group Oral daily administration Dose: 1 × 10^9^ CFU/day. Treatment length: 16 w.	↓ Liver fat accumulation ↓ Liver FFA content ↓ Liver inflammation ↓ Liver ALT and AST levels	Down-regulation of lipogenic markers in the liver: ↓ Protein expression of FAS and SREBP-1. ↑ Phosphorylation of ACC. Up-regulation of energy yielding pathways in the liver: ↑ Phosphorylation of AMPK. Down-regulation of oxidative stress in the liver: ↓ Content of MDA. ↑ Protein expression of HO-1 andNrf2. ↑ Content of CAT. ↑ Activity of SOD.
[18]	Male SPF C57BL/6J mice 6-week-old	Normal or Western diet (42% energy from fat).	*L. bulgaricus* *L. casei* *L. helveticus* *P. pentosaceus* KID7 Daily administration Dose: 1 × 10^9^ CFU/g suspended in distilled water. Treatment length: 8 w.	↓ Liver steatosis grade (all treated groups) ↓ Liver inflammation (all treated groups except animals receiving *L. casei*) ↓ Liver/bw ratio (groups treated with *L. bulgaricus*, *L. helveticus* and *P. pentosaceus*) ↓ Liver AST levels (groups treated with *L. bulgaricus* and *L. helveticus*) ↓ Liver ALT levels (group treated with *L. bulgaricus*) ↓ NAS (groups treated with *L. bulgaricus*, *L. helveticus* and *P. pentosaceus*)	Down-regulation of macrophage markers in the liver: ↓ Expression of *Cd68* (groups treated with *L. bulgaricus*, *L. helveticus*, *L. casei* and *P. pentosaceus*). Modulation of gut microbiota composition: ↓ *F*/*B* ratio (groups treated with *L. bulgaricus*, *L. helveticus*, *P. pentosaceus* and *L. casei*). ↑ Content of *A. muciniphila* (groups treated with *L. bulgaricus*, *L. helveticus* and *L. casei*). Down-regulation of pro-inflammatory markers and mediators in the liver: ↓ Gene expression of *Tnfα Il-6* and *Il-1β* (in all the treated groups).
[19]	Male Swiss mice 4-week-old	HFD (61% energy from fat).	*B. longum* Daily oral gavage Dose: 5 × 10^9^ CFU/kg bw/d Treatment length: 4 w.	↓ Liver lipid droplet size	Up-regulation of RAS related genes in the liver: ↑ Gene expression of *Ace2* and *Masr*.
[20]	Male C57BL/6N mice 6-week-old	HFD. Animals also received a 10% fructose solution.	*L. fermentum*—CQPC06 *L. delbrueckii* subsp. *Bulgaricus*—LDSB Daily oral gavage Dose of 1 × 10^9^ CFU/kg bw/d (*L. fermentum*—CQPC06) or 1 × 10^10^ CFU/kg bw/d (*L. fermentum*—CQPC06 and *L. delbrueckii* subsp. *Bulgaricus*—LDSB) suspended in sterile saline. Treatment length: 8 w.	↓ Liver weight and index ↓ Liver TG ↓ Serum and liver AST and ALT levels ↓ Serum and liver AKP levels	Decreased ROS levels in the livers of animals receiving *L. fermentum*—CQPC06 (at both doses) and *L. delbrueckii* subsp. *Bulgaricus*—LDSB. Up-regulation of fatty acid oxidation markers in the liver (*L. fermentum*—CQPC06 (at both doses)): ↑ Gene expression of *Cpt1* and *Ppar-α*. ↑ Protein expression of CPT1 and PPAR-α. Down-regulation of lipogenic markers in the liver (*L. fermentum*—CQPC06 (at both doses)): ↓ Gene expression of *C*/*ebp-α* and *Ppar-γ*. ↓ Protein expression of C/EBP-α and PPAR-γ. Up-regulation of markers of intestinal mucosa integrity (*L. fermentum*—CQPC06 (at both doses)): ↑ Protein expression of ZO-1, Occludin and Claudin-1. Modulation of gut microbiota composition (*L. fermentum*—CQPC06 (at both doses) and *L. delbrueckii* subsp. *Bulgaricus*—LDSB): ↓ *F*/*B* ratio. ↑ Content of *Akkermansia*.
[21]	Female C57BL/6 mice	WSD (40% energy from fat).	*L. rhamnosus GG* ATCC 53103 *L. lactis* subsp. *cremoris* ATCC 19257 Oral gavage Thrice weekly Dose: 1 × 10^9^ CFU Treatment length: 16 w.	↓ Liver weight (group treated with *L. cremoris*) ↓ Liver PC, PS, TG and TG content (group treated with *L. cremoris*) ↓ Liver lipid droplet area (group treated with *L. cremoris*) ↓ Liver inflammation (group treated with *L. cremoris*)	Down-regulation of hepatic content of lipids related to pro-inflammatory response: ↓ Levels of ARA containing lipids (group treated with *L. cremoris*). Down-regulation of inflammation associated metabolites in the liver: ↓ Levels of Resolvin E1, 9-HETE and 9HpODE (group treated with *L. cremoris*).
[22]	Male C57BL/6J mice 6-week-old	HFD (45% energy from fat).	*L. reuteri* 6475 *L. reuteri* VPL3461 Daily oral gavage (in a volume of 100 μL) Dose of 1 × 10^9^ CFU/mL Treatment length: 8 w.	↓ Liver TG content (all groups)	Not specified.
[23]	Male C57BL/6N mice 3–4-weeks-old	HFD (42% energy from fat).	*L. rhamnosus GG* Daily administration Dose: 1 × 10^8^ CFU/day mixed in the experimental diet. Treatment length: 17 w.	↓ Liver weight ↓ Liver TG content	Modulation of SCFA levels in the cecum: ↑ Acetate levels. Modulation of SCFA in the liver: ↑ Acetate levels. Modulation of anti-inflammatory lipid mediator levels: ↓ ώ6/ώ 3 PUFA ratio.
[37]	Male C57BL/6N mice 8-week-old	HFD/F (65% energy from fat and 30% dietary volume provided as fructose solution).	*L. plantarum* NA136 Daily oral administration daily Dose: 1 × 10^9^ CFU/day. Treatment length: 16 w.	↓ Liver lipid content	Modulation of gut microbiota composition: ↑ Bacterial richness and diversity. Up-regulation of intestinal mucosa integrity markers: ↑ Protein expression of tight-junction markers (ZO-1, Occludin, Claudin-1). ↓ Protein expression mucosal dysfunction markers (HIF-1α). Decreased serum levels of pro-inflammatory cytokines (TNF-α, IL-6, and IL-1β) and LPS. Down-regulation of pro-inflammatory markers and mediators in the liver: ↓ Protein expression of NF-κB. ↓ Phosphorylation of p38.
[24]	Male SPF C57BL/6J mice 6-week-old	WSD (42% energy from fat).	*L. acidophilus* *L. fermentum* *L. paracasei* *L. plantarum* Daily administration Dose: 1 × 10^9^ CFU suspended in drinking water. Treatment length: 8 w.	↓ Liver steatosis score (groups treated with *L. paracasei*, *L. plantarum* and *L. acidophilus*) ↓ Liver TG content (groups treated with *L. acidophilus*, *L. fermentum* and *L. paracasei*)	Modulation of microbiota composition: ↑ *Bacteroidetes* content (group treated with *L. paracasei*). ↓ *Firmicutes* content (group treated with *L. paracasei*).
[25]	Male Sprague-Dawley rats	HFD	*L. acidophilus* CGMCC 2106. *B. longum* CGMCC 2107. Daily administration Dose: 1 × 10^10^ CFU/mL suspended in drinking water. Treatment length: 12 w.	↓ Liver fat accumulation (group treated with *B. longum*)	Modulation of fecal microbiota composition: ↑ Bifidobacterium content (group treated with *B. longum*). ↑ Lactobacillus content (group treated with *L. acidophilus*).
[36]	Male Fischer 344 rats	CDAA diet (30% energy from fat). Animals were fed *ad libitum* and had free access to drinking water during the whole experiment.	*C. butyricum* Daily administration Dose: 8.5 × 10^9^ CFU/g mixed in the diet. Treatment length: 42 w.	↓ Liver total lipid and TG content ↓ Liver inflammation ↓ NAFLD progression (fibrosis) ↓ Serum ALT levels ↓ Liver lipid peroxidation ↓ Oxidative stress	Up-regulation of energy yielding pathways in the liver: ↑ Phosphorylation of AMPK. Up-regulation of fatty acid oxidation markers in the liver: ↑ Protein expression of PPARα. Down-regulation of lipogenic markers in the liver: ↓ Protein expression of SREBP-1c and PPAR-γ. Down-regulation of pro-inflammatory markers and mediators in the liver: ↓ Protein expression of NF-kB and TNF-α. Down-regulation of lipid peroxidation markers in the liver: ↓ Content of 4-HNE and MDA. Up-regulation of antioxidant markers in the liver: ↑ Protein expression of Nrf2 and HO-1. Up-regulation of markers of intestinal mucosa integrity: ↑ Protein expression of ZO1 and Ocln.
[26]	Male Sprague-Dawley rats	HFD.	*L. plantarum* NCU116-L *L. plantarum* NCU116-H Daily administration Dose: 1 × 10^8^ CFU/mL (*L. plantarum* NCU116-L) or 1 × 10^9^ CFU/mL (*L. plantarum* NCU116-H) suspended in a sterile saline solution. Treatment length: 5 w.	↓ Liver AST levels (group treated with *L. plantarum* NCU116-H) ↓ Liver oxidative stress ↓ Liver TC and TG content ↓ Liver inflammation	Down-regulation of oxidative stress markers in liver: ↓ MDA content (group treated with *L. plantarum* NCU116-H). Up-regulation of antioxidant markers in the liver: ↑ Activity of SOD and GPx (all groups). ↑ Activity of CAT (group treated with *L. plantarum* NCU116-H). ↑ T-AOC (all groups). Down-regulation of serum pro-inflammatory cytokines: ↓ Levels of LPS and IL-6 (all groups). ↓ Levels of TNFα (*L. plantarum* NCU116-H). Up-regulation of fatty acid oxidation and lipolysis markers in the liver: ↑ Gene expression of *Pparα*, *Pparγ*, *Pparδ*, *Pgc1α* and *Cpt1α* (all groups). Down-regulation of lipogenic markers in the liver: ↓ Gene expression of *Fas*, *Acc* and *Scd1* (all groups). Modulation of colonic microbiota composition: ↓ Gene expression of *Bacteroides* (all groups). ↑ Gene expression of *Lactobacillus* spp. and *Bifidobacterium* spp. (all groups).
[24]	Male Wistar rats	HFD (60% energy from fat).	*L. paracasei* Jlus66 Daily oral administration Doses: 1, 2 or 4 × 10^10^ CFU/d. Treatment length: 20 w.	↓ Liver weight ↓ Liver fat accumulation ↓ Liver inflammation ↓ Serum ALT levels (high dose)	Not specified.
[35]	Male Sprague-Dawley Rats 42-day-old	STD plus 20% fructose in drinking water.	*L. acidophilus* *B. coagulans* *L. casei* *L. reuteri* Daily administration Dose: 1 × 10^9^ CFU/mL suspended in drinking water. Treatment length: 16 w.	↓ Liver TG content (groups treated with *L. acidophilus* and *L. reuteri*) ↓ Serum ALT levels (all groups) ↓ Liver oxidative stress (all groups)	Up-regulation of antioxidant response in the liver: ↑ Content of glutathione (groups treated with *L. acidophilus* and *L. casei*). ↓ Liver ROS formation (groups treated with *L. acidophilus*, *L. casei* and *B. coagulans*). ↓ Liver protein-carbonylation (all groups). ↓ Liver lipid peroxidation (all groups).
[28]	Sprague-Dawley rats 8-week-old	HFD 54% energy from fat). Animals were injected with 600 mg/kg/day of D-galactose daily.	*L. fermentum* DR9 *L. plantarum* DR7 *L. reuteri* 8513d Daily administration Dose: 1 × 10^10^ CFU/day dissolved in 100 μL of saline and mixed into 1 g of experimental diet. Treatment length: 12 w.	↓ Liver lipid content (groups treated with *L. fermentum* DR9, *L. plantarum* DR7 and *L. reuteri* 8513d) ↓ Liver inflammation (groups treated with *L. fermentum* DR9, *L. plantarum* DR7 and *L. reuteri* 8513d) ↓ Liver ALP content (groups treated with *L. fermentum* DR9 and *L. plantarum* DR7)	Down-regulation of lipogenic markers in the liver: ↓ Gene expression of *Scd1* gene expression (groups treated with *L. fermentum* DR9 and *L. plantarum* DR7). Decreased liver content of pro-inflammatory cytokines: ↓ IL-4 levels (groups treated with *L. fermentum* DR9 and *L. plantarum* DR7). Up-regulation of energy yielding pathways in the liver: ↑ Gene expression of *Ampkα1* (groups treated with *L. fermentum* DR9 and *L. plantarum* DR7) and *Ampkα2* gene expression (group treated with *L. plantarum* DR7).
[16]	Male Wistar Rats 6-week-old	HFD (45% of energy from fat). Animals also received 10% fructose in drinking water.	*L. Plantarum* strain ATG-K2 *L. Plantarum* strain ATG-K6 Daily oral gavage Dose: 5 × 10^8^ CFU/d. Treatment length: 8 w.	↓ Liver TG and TC content ↓ Serum AST and ALT levels (all groups) ↓ Serum ALP levels (all groups) ↓ Liver lipid peroxidation	Down-regulation of lipogenic markers in the liver: ↓ Gene expression of *Srebp-1c* and *Fas* (all groups). ↓ Protein expression of SREBP-1c (group treated with *L. Plantarum* strain ATG-K6). ↓ Protein expression of FAS (all groups). ↓ Protein expression of C/EBP (group treated with *L. Plantarum* strain ATG-K2). ↑ Phosphorylation of ACC (group treated with *L. Plantarum* strain ATG-K2). Up-regulation of energy yielding pathways in the liver: ↑ Phosphorylation of AMPK (group treated with *L. Plantarum* strain ATG-K2). Up-regulation of fatty acid oxidation markers in the liver: ↑ Protein expression of CPT-1 (group treated with *L. Plantarum* strain ATG-K2). Decreased liver MDA content. Modulation of gut microbiota composition: ↓ Relative abundance of *Firmicutes* (all groups). ↑ Relative abundance of *Bacteroidetes* (all groups).
[30]	Male Wistar rats	HFD (60% energy from fat).	*B. animalis* subsp. *Lactis* V9 Daily oral gavage Dose: 1 × 10^9^ CFU/mL. Treatment length: 4 w.	↓ Liver TG and FFA content ↓ Serum AST and ALT levels ↓ Liver inflammation ↓ Progression to NASH	Down-regulation of lipogenic markers in the liver: ↓ Gene expression of *Srebp-1c* and *Fas.* Up-regulation of fatty acid oxidation markers in the liver: ↑ Gene expression of *Pparα*. Up-regulation of energy yielding pathways in the liver: ↑ Phosphorylation of AMPK. Down-regulation of NASH progression markers in the liver: ↓ Gene expression of *Nlrp3*, *Asc*, *Tlr-4* and *Tlr-9*. Down-regulation of pro-inflammatory markers and mediators in the liver: ↓ Gene expression of *Tnfα*, *IL-1β* and *IL-6*. ↓ Phosphorylation of JNK, NF-kB, ERK and AKT.
[31]	Male Sprague-Dawley rats	HFD (45% energy from fat).	*Eosinophil-Lactobacillus* Daily oral gavage (312 mg/kg). Dose: 1 × 10^7^ CFU/g. Treatment length: 8 w.	↓ Liver lipid content ↓ Liver inflammation ↓ Serum and liver ALT and AST levels	Modulation of gut microbiota composition: ↑ Bacterial diversity. ↓ Pathogenic bacteria content. Up-regulation of liver lipogenesis inhibitors: ↑ Protein expression of FGF15.

ACC: acetyl-CoA carboxylase; ACE2: angiotensin-converting enzyme 2; Akt: protein kinase B; ALP: alkaline phosphatase; ALT: alanine transaminase; AMPK: AMP-activated protein kinase; ApoB100: apolipoprotein B100; ARA: arachidonic acid; ASC: Apoptosis-associated speck-like protein containing a caspase recruitment domain; AST: aspartate transaminase; bw: body weight; d: day; CAT: catalase; CD36: cluster of differentiation 36; CD11b: cluster of differentiation molecule 11B; CD68: cluster of differentiation 68; CDAA: choline-deficient/L-amino acid-defined; C/EBP-α: CCAAT/enhancer binding protein α; CFU: colony-forming unit; ChREBP: carbohydrate-responsive element-binding protein; CPT1: carnitine palmitoyltransferase 1; CXCL10: C-X-C Motif Chemokine Ligand 10; ERK: extracellular-signal-regulated kinase; F4/80: EGF-like module-containing mucin-like hormone receptor-like 1; FAS: fatty acid synthase; F/B: *Firmicutes*/*Bacteroidetes*; FGF15: fibroblast growth factor-15; FGF21: fibroblast growth factor-21; GPx: glutathione peroxidase; HFD: high-fat diet; HFD/F: high-fat and fructose diet; HIF-1α: hypoxia Inducible factor 1 Subunit α; HO-1: heme oxygenase 1; IL-1β: interleukin 1β; IL-4: interleukin 4; IL-6: interleukin 6; IL-8R: interleukin 8 receptor; IL-12: interleukin 12; JNK: janus kinase; LDL-c: LDL cholesterol; LPS: lipopolysaccharide; MAO: monoamino oxidase; MASR: Mas receptor; MDA: malondialdehyde; NAS: NAFLD activity score; NASH: non-alcoholic steatohepatitis; NF-κB: nuclear factor kappa B; NLRP3: nod-like receptor protein 3; Nrf2: nuclear factor erythroid 2–related factor 2; Ocln: Occludin; p38: p38 MAP kinase; PBS: phosphate buffered saline; PGC1α: peroxisome proliferator-activated receptor gamma coactivator 1-α; PP2AC: protein phosphatase 2 catalytic subunit α; PPAR-α: peroxisome proliferator-activated receptor α; PPAR-γ: peroxisome proliferator-activated receptor γ; PPAR-δ: peroxisome proliferator-activated receptor δ; PUFA: polyunsaturated fatty acids; RAS: renin–angiotensin system; ROS: reactive oxygen species; SCD1: stearoyl-CoA desaturase; SCFA: short-chain fatty acids; SOD: superoxide dismutase; SPF: specific pathogen-free; SREBP-1: Sterol regulatory element-binding protein 1; STD: standard; T-AOC: total antioxidant capacity; TC: total cholesterol; TG: triglycerides; TLR-4: toll-like receptor 4; TLR-9: toll-like receptor 9; Tnfα: tumor necrosis factor α; w: weeks; WSD: western-style diet; ZO1: Zonula Occludens 1; 4-HNE: 4-hydroxynonenal; 9-HETE: 9-hydroxy-5Z,7E,11Z,14Z-eicosatetraenoic acid; 9HpODE: 9-hydroperoxy-10E,12Z-octadecadienoic acid; ↓: significant reduction; ↑: significant increase.

**Table 2:** Preclinical studies (rodent models) addressing the effects of different probiotic mixtures in diet-induced NAFLD.

Reference	Animal Model	Experimental Conditions	Probiotic Treatment	Effects in Liver	Mechanisms of Action
[44]	Male C57BL/6J mice 5-week-old	HFD (60% energy from fat).	Probiotic mixtures: Bacillus mixture: *B. sonorensis JJY12–3*, *B. paralicheniformis JJY12–8*, *B. sonorensis JJY13–1*, *B. sonorensis JJY 13–3* and *B. sonorensis JJY 13–8*. VSL#3: *L. acidophilus*, *L. plantarum*, *L. casei*, *L. delbrueckii subspecies bulgaricus*, *B. breve*, *B. longum*, *B. infantis* and *S. salivarius* subspecies *thermophilus* Daily administration Dose of 1 × 10^8^ CFU/day Treatment length: 13 w.	↓ Liver weight (group treated with *Bacillus* mixture) ↓ Liver fat accumulation (group treated with *Bacillus* mixture) ↓ Liver TG content (group treated with *Bacillus* mixture) ↓ Liver inflammation (group treated with *Bacillus* mixture)	Up-regulation of markers related to fatty acid oxidation in the liver (group treated with *Bacillus* mixture): ↑ Gene expression of *Acox1* and *Cpt1*. ↑ Protein expression of PCG1α. Down-regulation of pro-inflammatory markers and mediators in the liver (group treated with *Bacillus* mixture): ↓ Gene expression of *Tnfα*, I*nfγ*, *Mcp-1* and *Il-12*. Up-regulation of markers of intestinal mucosa integrity (group treated with *Bacillus* mixture): ↑ Gene expression of *Zo1* and *Ocln*. ↑ Protein expression of Occludin. Modulation of cecum SCFA content and hepatic receptors: ↓ Cecum acetate levels (group treated with *Bacillus* mixture). ↓ Gene expression of acetate receptor *Gpr43* (group treated with *Bacillus* mixture).
[45]	Male C57BL/6 mice 6-week-old	HFD (60% energy from fat).	Probiotic mixture: *L. plantarum* LC27 *B. longum* LC67 Daily oral gavage 3:1 proportion (0.75 × 10^9^ CFU of *L. plantarum* and 0.25 × 10^9^ CFU of *B. longum*) Treatment length: 4 w.	↓ Liver weight ↓ Liver lipid accumulation ↓ Liver TG content (group treated with *L. plantarum* LC27) ↓ NAS (all groups) ↓ Serum AST and ALT levels	Down-regulation of pro-inflammatory markers and mediators in the liver: ↓ Levels of TNFα. ↓ Activity of MPO. ↓ Protein expression of iNOS and COX-2. ↓ Protein expression of p65. ↓ Phosphorylation of p65. ↑ Protein expression of Iκβα. ↓ Phosphorylation of Iκβα. Up-regulation of energy yielding pathways in the liver: ↑ Phosphorylation of AMPK. Down-regulation of periportal fibrogenesis markers in the liver: ↓ Protein expression of α-SMA. ↓ Phosphorylation of α-SMA. Up-regulation of markers of intestinal mucosa integrity: ↑ Protein expression of Occludin-1 and Claudin-1. Modulation of gut microbiota composition: ↓ Proportion of *Firmicutes*, *Bacteroidetes*, *Proteobacteria*, *Deferribacteria*. ↓ *F*/*B* ratio. ↓ *P*/*B* ratio. ↑ Proportion of *Actinobacteria*. ↓ Gut content of LPS.
[46]	Male C57BL/6N mice >78-week-old	HFD (60% energy from fat).	Human origin probiotic mixture: 5 strains of *Lactobacillus* 5 strains of *Enterococcus* Daily administration Dose: 1 × 10^9^ CFU/mL suspended in the drinking water. Treatment length: 10 w.	↓ Liver fat accumulation ↓ Liver inflammation	Enhanced microbial diversity enriched in beneficial commensal bacteria. Up-regulation of markers of intestinal mucosa integrity: ↑ Gene expression of *Zo1* and *Ocln*. Modulation of microbial metabolites in the gut: ↑ Abundance of butyrate and propionate.
[18]	Male SPF C57BL/6J mice 6-week-old	Normal or a Western diet (42% energy from fat).	Probiotic mixtures: Mix 1: *L. casei* + *L. helveticus* Mix 2: *L. casei* + *L. helveticus* + *P. pentosaceus* KID7 Mix 3: *L. casei* + *L. helveticus* + *L. bulgaricus* Daily administration Dose of 1 × 10^9^ CFU/g suspended in distilled water. Treatment length: 8 w.	↓ Liver steatosis grade (all groups) ↓ Liver inflammation (all groups) ↓ Liver/bw ratio (groups treated with Mix 1 and Mix 2) ↓ NAS (all groups)	Down-regulation of pro-inflammatory markers and mediators in the liver: ↓ Gene expression of *Tnfα* (all groups). ↓ Gene expression of *Il-6* (groups treated with Mix 2 and Mix 3). ↓ Gene expression of *Il-1β* (group treated with Mix 3).
[47]	Male SPF C57BL/6J mice 3–4-week-old	HFD (60% energy from fat).	Probiotic mixture: *L. plantarum* KLDS1.0344 *L. plantarum* KLDS1.0386 Daily oral gavage Dose: 10^8^ CFU Treatment length: 8 w.	↓ Serum ALT and AST levels ↓ Liver TG content	Down-regulation of oxidative stress markers in the liver: ↓ Content of MDA. ↑ Content of GSH-Px. ↑ Content of CAT. ↑ Content of SOD. Modulation of gut microbiota composition: - Relative abundance of *Parabacteroides*, *Eubacterium xylanophilum group*, *GCA-900066575*, *Lachnoclostridium*, *Lachnospiraceae UCG-006* and *Rombustia*. ↑ Richness of *Lachnospiraceae NK4A136 group* and *Bacteroides*. Modulation of gut SCFA production: ↑ Levels of acetic and butyric acids.
[48]	Male C57BL/6J mice 5-week-old	HFD (60% energy from fat).	Probiotic mixture: *B. subtilis* (1.4 × 10^9^ CFU) *E. faecium* (1.55 × 10^10^ CFU) Daily oral gavage. Treatment length: 16 w.	↓ Liver index ↓ Serum ALT and AST levels ↓ Liver lipid accumulation	Up-regulation of fatty acid oxidation markers in the liver: ↑ Protein expression of CPT1 and PPARα. Down-regulation of pro-inflammatory markers and mediators in the liver: ↓ LPS levels. ↓ Gene expression of *Il-1β*, *Il-6* and *Tnf-α*. ↓ Protein expression of TLR-4 and NF-κβ. Up-regulation of markers of intestinal mucosa integrity: ↑ Protein expression of Occludin-1 and Claudin-1. Modulation of gut microbiota composition: ↓ *F*/*B* ratio. ↓ Relative abundance of *Firmicutes*. ↑ Relative abundance of *Bacteroidetes*, *Verrucomicrobia*, *Akkermansia* and *Oscillibacter*.
[49]	Male Zucker-Lepr*^fa^*^/*fa*^ rats	Chow diet.	Probiotic mixture: *L. paracasei* CNCM I-4034 *B. breve* CNCM I-4035 Daily oral gavage Dose: 10^10^ CFU. Treatment length: 30 days.	↓ Liver TG content	Decreased serum levels of pro-inflammatory markers cytokines and mediators: ↓ Levels of TNF-α. ↓ Levels of LPS.
[50]	Male albino rats 6-week-old	HFSD (59% energy from fat).	Probiotic mixture: *L. acidophilus* (10 × 10^8^ CFU/g). *L. plantarum* (9.8 × 10^7^ CFU/g). *B. bifidum* (2 × 10^6^ CFU/g). *B. subtilis* fermentation extract (50 g per kg of product). *A. oryzae* fermentation extract (50 g per kg of product). Daily administration Dose: 1 g of probiotic mixture/kg diet Treatment length: 4 w.	↓ Serum ALT levels ↓ NAS ↓ Liver inflammation	Not specified.
[51]	Male Sprague-Dawley rats 7-week-old	HCD (15% energy from fat).	Probiotic mixture: *B. longum CBG-C11* *B. lactis* CBG-C10 *B. breve* CBG-C2 *L. reuteri* CBG-C15 *L. plantarum* CBG-C21 Daily by oral gavage Doses: Low dose (1.65 × 10^9^ CFU/kg/d) Medium dose (5.5 × 10^9^ CFU/kg/d) High dose (1.65 × 10^10^ CFU/kg/d) Treatment length: 8 w.	↓ Hepatic steatosis score (group treated with the high dose) ↓ Liver TG and TC content (all groups) ↓ Liver AST and ALT levels (all groups)	Down-regulation of lipogenic markers in the liver: ↓ Protein expression of FAS, ACC and SREBP-1 (all doses).
[35]	Male Sprague-Dawley rats 42-day-old	STD + 20% fructose in drinking water.	Probiotic mixture: *L. acidophilus* *B. coagulans* *L. casei* *L. reuteri* Daily administration Dose: 1 × 10^9^ CFU/mL suspended in drinking water. Treatment length: 16 w.	↓ Liver TG content ↓ Serum ALT levels ↓ Liver oxidative stress	Up-regulation of antioxidant response in the liver: ↑ Content of glutathione. ↓ Liver ROS formation. ↑ Liver total antioxidant level. ↓ Liver protein-carbonylation. ↓ Liver lipid peroxidation.

ACC: acyl-CoA-carboxylase; Acox 1: acyl-CoA oxidase 1; ALT: alanine transaminase; AMPK: AMP-activated protein kinase; α-SMA: smooth muscle alpha-actin; bw: body weight; CAT: catalase; CFU: colony-forming unit; COX-2: cyclooxygenase-2; CPT1: carnitine palmitoyltransferase 1; FAS: fatty acid synthase; F/B: *Firmicutes*/*Bacteroidetes* ratio; GPR-43: G-protein coupled receptor 43; GSH-Px: plasma glutathione peroxidase; HCD: high-cholesterol diet; HFD: high-fat diet; HFSD: high-fat sucrose diet; Iκβα: NF-κβ inhibitor; IL-1β: interleukin 1β; IL-2: interleukin 2; IL-6: interleukin 6; IL-12: interleukin 12; INFγ: interferon γ; iNOS: inducible nitric oxide synthase; LPS: lipopolysaccharides; MCP-1: monocyte chemoattractant protein-1; MDA: malonaldehyde; MPO: myeloperoxidase; NAS: NAFLD activity score; NF-κβ: nuclear factor kappa-light-chain-enhancer of activated B cells; Ocln: Occludin; PBS: phosphate buffered saline; P/B: Proteobacteria/Bacteroidetes ratio; PCG1α: peroxisome proliferator-activated receptor gamma coactivator 1-α; PPARα: peroxisome proliferator-activated receptor alpha; ROS: reactive oxygen species; SCFA: short-chain fatty acids; SOD: superoxide dismutase; SPF: specific pathogen-free; SREBP-1: sterol regulatory element-binding transcription factor-1; STD: standard; TG: triglycerides; TLR-4: toll-like receptor 4; Tnfα: tumor necrosis factor α; w: weeks; ZO-1: Zonula Occludens; ↓: significant reduction; ↑: significant increase.

**Table 3:** Studies conducted in humans addressing the effects of different probiotics (single strain and mixtures) in NAFLD treatment (PICO format).

Reference	Population	Intervention	Comparison	Outcome
[53]	28 adults with NAFLD 20 men 8 women.	Daily consumption Probiotic mixture (5 × 10^8^ CFU): *L. bulgaricus* *S. thermophilus* Treatment period: 3 months.	Placebo.	↓ Serum ALT, AST and GGT levels.
[59]	72 obese adults with NAFLD 33 men 39 women Age: 23–63 years old. BMI: 25–40 kg/m^2^	Daily consumption Probiotic-enriched yogurt (300 g/d): *L. bulgaricus* *S. thermophilus* *L. acidophilus* (6.46 × 10^6^ CFU/g) *B. lactis Bb12* (4.97 × 10^6^ CFU/g) Treatment period: 8 weeks	Daily consumption Conventional yogurt (300 g/d) *L. bulgaricus* *S. thermophilus*	↓ Serum ALT and AST levels.
[54]	64 obese adolescents with NAFLD Age: 10–18 years old BMI >85th percentile (age and sex specific)	Daily consumption Probiotic mixture (1 capsule): *L. acidophilus* ATCC B3208 (3 × 10^9^ CFU) *B. lactis* DSMZ 32269 (6 × 10^9^ CFU) *B. bifidum* ATCC SD6576 (2 × 10^9^ CFU) *L. rhamnosus* DSMZ 21690 (2 × 10^9^ CFU) Treatment period: 12 weeks	Placebo.	↓ Serum ALT and AST levels. ↓ Fatty liver grade (sonographic grading).
[55]	58 adult patients with NAFLD and T2DM Age: 18–65 years old BMI > 25 kg/m^2^	Daily consumption Probiotic mixture (1 sachet of 10 g). *Lactobacillus + Lactococcus* (6 × 10^10^ CFU/g) *Bifidobacterium* (1 × 10^10^ CFU/g) *Propionibacterium* (3 × 10^10^ CFU/g) *Acetobacter* (1 × 10^6^ CFU/g) Treatment period: 8 weeks	Placebo.	↓ FLI and LS. ↓ Serum AST and GGT levels. Decreased circulating levels of proinflammatory markers: ↓ Serum TNF-α and IL-6 levels.
[56]	65 obese adults with NAFLD 33 men 32 women Age: 19–75 years old BMI >25 kg/m^2^ Mean hepatic MRI-PDFF 16.2%	Daily consumption Probiotic mixture (containing 1 × 10^9^ CFU/1.4 g) *L. acidophilus* CBT LA1 *L. rhamnosus* CBT LR5 (human feces) *L. paracasei* CBT LPC5 (Korean fermented food—jeotgal) *P. pentosaceus* CBT SL4 (Korean fermented vegetable product—kimchi) *B. lactis* CBT BL3 *B. breve* CBT BR3 (Korean infant feces) Treatment period: 12 weeks	Placebo.	↓ IHF fraction. Modulation of gut microbiota composition: ↑ Relative abundances of *L. acidophilus* *L. rhamnosus* *P. pentosaceus* *B. lactis* *B. breve*
[57]	30 adults with NAFLD: [ALT] and [AST] >1.5-fold normal levels	Daily consumption Probiotic mixture (2 capsules containing 11.25 × 10^10^ CFU, each) *L.* paracasei DSM 24733, *L. plantarum* DSM 24730 *L. acidophilus* DSM 24735 *L. delbrueckii* subsp. *Bulgaricus* DSM 24734 *B. longum* DSM 24736 *B. infantis* DSM 24737 *B. breve* DSM 24732 *S. thermophilus* DSM 24731 Treatment period: 12 months	Placebo.	Improvement of liver histology: ↓ Hepatocyte ballooning. ↓ Lobular inflammation. ↓ NAS score. ↓ Serum ALT levels. ↓ Serum ALP levels. ↓ Serum pro-inflammatory cytokines: Il-1β, IL-6 and TNF-α.
[58]	60 adults with NAFLD 43 men 17 women Age: 20–60 years old BMI: 20–40 kg/m^2^	Daily consumption Probiotic mixture (1 capsule containing 5 × 10^9^ CFU) *L. casei* *L. rhamnosus* *L. acidophilus* *B. longum* *B. breve* Treatment period: 12 weeks	Placebo.	↓ Serum ALT, AST and GGT levels ↓ Serum ALP levels.
[60]	35 adults with NAFLD 28 men 7 women Age: 25–70 years old Mean BMI 32.6 ± 5.0 kg/m^2^	Daily consumption Probiotic mixture (2 sachets of VSL#3 twice daily) *S. thermophilus* *B. breve* *B. infantis* *B. longum* *L. acidophilus* *L. plantarum* *L. paracasei* *L delbrueckii* subsp. *bulgaricus* Treatment period: 10 weeks	Placebo.	No significant improvements in markers of liver injury.
[61]	39 obese adults 28 men 11 women with NAFLD: Fatty liver score >263 dB/m	Daily consumption Probiotic mixture (1 sachet containing 3 × 10^10^ CFU twice daily) *L. acidophilus* BCMC 12,130 (107 mg) *L. lactis* MCMC 12,451 (107 mg) *B. bifidum* BCMC 02290 (107 mg) *B. infantis* BCMC 02129 (107 mg) *B. longum* BCMC 02120 (107 mg) Treatment period: 6 months	Placebo.	No significant improvements in liver fibrosis parameters or serum markers of inflammation.

ALP: alkaline phosphatase; ALT: alanine transaminase; AST: aspartate aminotransferase; BMI: body mass index; CFU: colony-forming units; FLI: fatty liver index; GGT: γ glutamyl transferase; HbA1c: glycated hemoglobin; IHF: intrahepatic fat; IL-1β: interleukin 1β; IL-6: interleukin 6; m: men; LS: liver stiffness; MRI-PDFF: magnetic-resonance-imaging-derived proton density fat fraction; NAFLD: non-alcoholic fatty liver disease; NAS: NAFLD activity score; TNF-α: tumor necrosis factor α; w: women; ↓: significant reduction; ↑: significant increase.

## Data Availability

Not applicable.
